# Evidence-based Evaluation to Promote High-value Care in Pediatric Critical Care: It Is Time to Fully Adopt

**DOI:** 10.1097/pq9.0000000000000863

**Published:** 2025-12-23

**Authors:** Maya Dewan, Rajit K. Basu, Erin Bennett, Lily B. Glater-Welt, Mark Hall, Benson S. Hsu, Lia Lowrie, Venessa Lynn Pinto, Alexandre T. Rotta, Edward Seferian, Linda Siegel, Heather A. Wolfe, Vijay Srinivasan

**Affiliations:** * Division of Critical Care Medicine, Department of Pediatrics, Cincinnati Children’s Hospital Medical Center, University of Cincinnati College of Medicine, Cincinnati, Ohio; † Division of Critical Care Medicine, Department of Pediatrics, Ann & Robert Lurie Children’s Hospital of Chicago, Feinberg School of Medicine, Northwestern University, Chicago, Ill.; ‡ Division of Critical Care Medicine, Department of Pediatrics, University of Arkansas for Medical Sciences, Arkansas Children’s Hospital, Little Rock, Ark.; § Department of Pediatrics, Division of Critical Care, Zucker School of Medicine, Cohen Children’s Medical Center, New Hyde Park, N.Y.; ¶ Department of Pediatrics, Division of Critical Care Medicine, Nationwide Children’s Hospital, Columbus, Ohio; ∥ Department of Pediatrics, University of South Dakota Sanford School of Medicine, Sioux Falls, S.Dak.; ** Division of Critical Care Medicine, Department of Pediatrics, Saint Louis University School of Medicine, SSM Health Cardinal Glennon Children’s Hospital, St. Louis, Mo.; †† Division of Critical Care Medicine, Department of Pediatrics, Baylor College of Medicine, Texas Children’s Hospital, Houston, Tex.; ‡‡ Division of Pediatric Critical Care Medicine, Duke University Medical Center, Durham, N.C.; §§ Department of Anesthesiology and Critical Care Medicine, The Johns Hopkins Hospital, The Johns Hopkins University School of Medicine, Baltimore, Md.; ¶¶ Division of Anesthesiology and Critical Care Medicine, The Children’s Hospital of Philadelphia, University of Pennsylvania Perlman School of Medicine, Philadelphia, Pa; ∥∥ Division of Pediatric Critical Care Medicine, Department of Pediatrics, Children’s Healthcare of Atlanta, Emory University School of Medicine, Atlanta, Ga.

## Abstract

**Introduction:**

Evidence-based recommendations for high-value care in pediatric critical care are needed. We sought to develop recommendations to identify unnecessary interventions, enhance outcomes, and promote efficient, patient-centered practices.

**Methods:**

Using a modified Delphi process, a multi-institutional panel of pediatric intensivists from a national pediatric critical care quality improvement group identified 30 potential topics and selected 20 high- and medium-priority topics for further evaluation, and narrowed them to 10 by consensus voting. A structured scoping review of literature published between 2014 and 2023 identified 10 practices, which were critically assessed using the PICOST framework. The panel selected 5 priority recommendations based on evidence strength, feasibility, and potential impact.

**Results:**

The panel developed 5 evidence-based recommendations: (1) prioritize early and progressive mobilization in the pediatric intensive care unit, (2) avoid reflexive culture testing in febrile children without clinical signs of infection, (3) conduct daily tracheal extubation readiness assessments to prevent unnecessary delays, (4) limit red blood cell transfusions in stable, nonbleeding patients with hemoglobin greater than 7 g/dL, and (5) initiate enteral nutrition within 48 hours when clinically safe and feasible. Each recommendation is grounded in current best evidence and aims to minimize harm while optimizing resource use and outcomes.

**Conclusions:**

Incorporating high-value care principles into pediatric critical care holds promise for reducing unwarranted variation, minimizing harm, and improving the efficiency and effectiveness of care. Future efforts should focus on implementation strategies, practice integration, and ongoing evaluation to sustain improvements and close the gap between evidence and adoption.

## INTRODUCTION

High-quality, evidence-based care in the pediatric intensive care unit (PICU) is essential for improving patient outcomes while minimizing unnecessary interventions, healthcare costs, and potential harm. Advances in pediatric critical care have improved survival rates,^1,2^ but they have also highlighted concerns about PICU-acquired morbidities, resource overuse, and variations in care.^3–5^ To ensure effective and efficient care, best practices that promote high-value care and reduce unnecessary interventions must be established.

High-value care in the PICU optimizes patient outcomes while minimizing waste and harm. This concept is well developed in adult critical care but remains underexplored in pediatrics.^6^ Many PICU practices, such as routine daily chest radiographs, reflexive culture testing, and deep sedation, persist despite a lack of strong evidence. Conversely, delays in early mobilization, enteral nutrition (EN), and readiness assessments for tracheal extubation can negatively impact recovery and length of stay. The challenge is distinguishing necessary interventions from those that do not improve outcomes and may cause harm.

This review synthesized current evidence to identify key pediatric critical care practices that should be encouraged, discouraged, or refined. The objectives are to

1. identify interventions that reduce delays and improve outcomes;
2. highlight practices that should be discontinued due to lack of benefit or harm;
3. establish essential practices to ensure optimal patient management.

By applying an evidence-based approach, pediatric intensivists can align care with high-value principles, improving efficiency, reducing costs, and enhancing the quality and safety of care for critically ill children.^7^

## METHODS

Key stakeholders were engaged using a modified Delphi method to select and create a panel of experts to achieve consensus. A 14-member multidisciplinary working group composed of pediatric critical care specialists with expertise in guideline development, improvement science, and value-based care was convened to revisit and update recommendations aligned with the Choosing Wisely campaign, specifically for pediatric critical care medicine. The group included members affiliated with the American Academy of Pediatrics (AAP) and the Society of Critical Care Medicine (SCCM). Although the Choosing Wisely campaign was formally sunset by the ABIM Foundation during the group’s work, the panel continued its efforts, maintaining a focus on identifying low-value practices and promoting high-value, evidence-based care in the PICU. The views expressed herein reflect the consensus of the working group and do not necessarily represent the official positions, policies, or endorsements of the AAP, SCCM, or the institutions with which the members are affiliated.

During a regularly scheduled meeting, the initial 14-member multidisciplinary team collaborated with members from the AAP and the SCCM Pediatric Quality Improvement Subgroup to generate a comprehensive list of potential recommendations. Through a brainstorming session, the group identified a total of 30 topics for consideration, as detailed in the supplemental table. (**See table, Supplemental Digital Content 1**, which displays the original 30 recommendations considered, https://links.lww.com/PQ9/A725.) Following topic generation, each of the 14 members individually evaluated the recommendations using a Research Electronic Data Capture survey. The survey used a scoring system: “must be included” (3 points), “should be included” (2 points), “consider inclusion” (1 point), and “do not include” (0 points). Based on the cumulative scores, topics were ranked and categorized into 3 priority groups—high, medium, and low—each containing 10 topics. To focus efforts on the most impactful practices, the low-priority topics were excluded from further consideration. This refinement resulted in a shortlist of 20 topics, as presented in Table 1. The group then conducted a consensus vote to further narrow the selections, ultimately identifying 10 topics for inclusion in the final recommendations.

**Table 1.** List of 20 Topics for Consideration from National Pediatric Critical Care Quality Improvement

Practices to Reduce Delays as May Improve Outcome	Practices to Discourage as May Worsen Outcome	Common Practices with Unclear Impact on Outcome
• Progressive mobility • Delirium screening • Enteral feeding • Discussion on goals of care • Routine deep vein thrombosis prophylaxis • ERT	• Routine culture practices • Routine electrolyte testing • Blood transfusions • Antibiotics in viral illness • Routine hypotonic fluid use • Routine gastrointestinal prophylaxis • Continuous sedation and neuromuscular blockade • Limiting bedside family presence	• Respiratory clearance treatments • Steroid use in shock • Empiric aspiration pneumonia coverage • Volume overload assessment • Plasma exchange indications • Isolation precautions

PubMed and MEDLINE were systematically searched from 2014 to 2023 using terms appropriate for each topic. An iterative process was used, with keywords from each article, to ensure all relevant and appropriate articles were included. In addition, the International Clinical Trials Registry Platform, the US Clinical Trials Registry, and Cochrane CENTRAL were searched to include any ongoing trials. Inclusion criteria were English-language studies of children aged 0–18 in the PICU or the pediatric cardiac intensive care unit.

Working groups—composed of 13 of the original 14 multidisciplinary members, with 1 member stepping away due to other responsibilities—were formed, and a scoping review methodology was used to comprehensively and systematically examine the relevant literature on the chosen topics. Additional articles, referenced in articles from 2014 to 2023 that were considered foundational, were also included. Each of the 10 topics was presented to the rest of the group using the PICOST framework (Population, Intervention, Comparison, Outcomes, Study design, Timeframe). After all topics were presented, the panel voted on the top 5 recommendations.

### Ethical Considerations

As this study was a review of published literature and did not involve direct patient data collection, institutional review board approval was not required.

### Evidence-based Recommendations

#### Prioritize Early Mobilization Therapy in the PICU

Structured, progressive, developmentally appropriate early mobilization therapy programs are safe in the PICU and may improve outcomes, including reduced mechanical ventilation duration, decreased delirium incidence, and preserved psychomotor function.

##### Supporting Evidence

Early mobilization therapy in critically ill patients is defined as psychomotor activities aimed at preventing and treating weakness associated with critical illness. Adult early mobilization programs reduce mechanical ventilation duration, shorten length of stay, and improve functional outcomes.^8^ In the PICU, implementation is more complex due to variability in age, diverse conditions, developmental and sleep needs, and the lack of standardized pediatric functional outcomes.^9^ Despite this, early mobilization programs can be safely implemented in the PICU without frequent device dislodgement or physiological instability.^10–13^ A prospective multicenter cohort study of ABCDEF bundle implementation, including early mobilization, demonstrated an association between greater bundle use and decreased mortality. However, increased bundle use was not associated with characteristics generally associated with decreased mortality, that is, shorter mechanical ventilation duration, shorter PICU length of stay, and lower delirium incidence.^14^ Several reports from single centers in specific disease subsets show improved functional outcomes.^15–17^

##### Clinical Implications

As focused attention on central line care has decreased hospital-acquired infections, every PICU will need to realign the focus and priorities of multidisciplinary personnel and resources toward the preservation and restoration of psychomotor function in the critically ill pediatric patient to speed patient and family recovery.

#### Do Not Reflexively Send Blood, Urine, or Tracheal Cultures in the Setting of Isolated Fever

Blood, urine, and tracheal cultures should not be obtained reflexively in pediatric ICU patients with isolated fever unless there is clinical suspicion of infection.

##### Supporting Evidence

Diagnostic stewardship efforts have demonstrated that indiscriminate culture testing leads to increased false-positive results, unnecessary antibiotic use, and increased hospital-acquired infections.^18,19^ There is wide variation in clinical practice regarding ordering cultures and/or starting antibiotics, and many PICU physicians report a low threshold for evaluating and treating fever in critically ill children.^20,21^ Clinical decision tools have been developed that thoughtfully target the elimination of low-value diagnostic testing in the setting of fever in a PICU patient, including blood culture,^22^ tracheal cultures,^23,24^ and a comprehensive algorithm for fever evaluation.^25^ Evaluation of the implementation of these tools has shown reductions in cultures sent and antibiotic use, without an increase in sepsis, mortality, or length of stay. A subsequent multicenter study found that implementing blood culture stewardship protocols reduced culture rates, antibiotic days, and central line–associated bloodstream infections without increasing mortality.^26,27^

##### Clinical Implications

Clinicians should base culture decisions on clinical assessment rather than routine fever workups. Standardized diagnostic stewardship protocols can safely guide culture ordering practices.

#### Perform a Daily Assessment of Extubation Readiness for All Mechanically Ventilated Children and Avoid Unnecessary Extubation Delays

A structured daily assessment of tracheal extubation readiness should be performed in all mechanically ventilated children, and tracheal extubation should not be delayed when readiness is demonstrated.

##### Supporting Evidence

Delays in tracheal extubation are associated with increased ventilator-associated complications and prolonged ICU stays. Daily protocolized screening for tracheal extubation readiness testing (ERT) supports timely identification of patients meeting physiological, ventilatory, or pathology-specific criteria for safe liberation from invasive mechanical ventilation (IMV).^28^ Evidence from randomized controlled trials (RCTs)^29–33^ and quality improvement studies^34–36^ demonstrate that systematic ERT screening shortens IMV duration, ranging from several hours to multiple days.^29,31,33,34^ Systematic ERTs may also lower tracheal extubation failure rates,^28,30^ though isolating the effect of screening from other bundle elements is challenging. Barriers such as increased workload and screening fatigue can be mitigated by embedding screening into routine clinical workflows.^17,23^ A structured ERT bundle has been associated with lower tracheal extubation failure rates (absolute risk reduction, 3.3%–11.7%).^28,34^ SBTs are a core ERT component and demonstrate high predictive value for tracheal extubation success (>90%),^30,37^ with some studies reporting up to 30% reduction in IMV duration.^29,34^

##### Clinical Implications

Standardizing ERT within PICU workflows reduces variability in tracheal extubation practices, promotes timely liberation from IMV, and minimizes the risk of tracheal extubation failure. Implementation barriers, such as excessive sedation and unstructured decision-making, should be addressed to optimize adherence and outcomes. Addressing these barriers can—and should—start at the local level, thereby increasing the ability to provide evidence for widespread strategies to mitigate them.

#### Do Not Transfuse Red Blood Cells in Hemodynamically Stable, Nonbleeding Pediatric ICU Patients with a Hemoglobin Greater Than 7 g/dL

Restrictive transfusion strategies should be applied in hemodynamically stable, nonbleeding, pediatric ICU patients, with a hemoglobin (Hgb) transfusion threshold of 7 g/dL.

##### Supporting Evidence

The Transfusion Requirements in Pediatric Intensive Care Unit (TRIPICU) study, a noninferiority RCT, showed that a restrictive red blood cell (RBC) transfusion strategy (hemoglobin threshold of 7 g/dL) was as safe as a liberal transfusion approach (hemoglobin >9.5 g/dL).^38^ There was no significant difference between groups in multiorgan dysfunction syndrome or mortality. Subsequent subgroup analyses for patients with stable sepsis, general pediatric surgery, and noncyanotic cardiac surgery confirmed these findings.^39–41^ A secondary analysis of noncardiac, hemodynamically stable patients in a different randomized trial (Age of Blood in Children in PICUs) showed that deferring transfusion until hemoglobin was less than 7 g/dL was not associated with increased organ dysfunction and was associated with increased survival to ICU discharge.^42^ The Pilot-Optimizing Transfusion Threshold in Critically Ill Children with Anemia study, a smaller multicenter, international, noninferiority pilot RCT to assess the feasibility of Optimizing Transfusion Threshold in Critically Ill Children with Anemia, similarly showed no differences in outcomes between restrictive versus liberal transfusion thresholds.^43^ There is insufficient evidence for a RBC transfusion recommendation in cyanotic congenital heart disease patients, with only 1 RCT comparing transfusion thresholds of 10.8 and 8 g/dL.^44^ In 2018, evidence-based consensus recommendations were published that recommended considering the overall clinical context, the risks, benefits, and alternatives to transfusion.^45,46^

##### Clinical Implications

A conservative approach to RBC transfusion may reduce transfusion-related risks such as fluid overload, sensitizations, transfusion reactions, and infections, as well as cost. Adopting a restrictive transfusion strategy is safe and can minimize unnecessary blood product usage. Transfusion decisions should be based on individual patient factors rather than routine hemoglobin thresholds.

#### Initiate EN Within 48 Hours When Safe and Feasible

Do not delay the initiation of EN beyond 48 hours in critically ill pediatric patients if it is safe and feasible.

##### Supporting Evidence

The provision of EN is associated with improved gastrointestinal integrity, reduced infection risk, and decreased mortality in critically ill children. Evidence from a small RCT,^47^ a secondary analysis of an RCT,^48^ and large cohort studies of critically ill children, including those with specific disease states^49–52^ and systematic reviews/meta-analyses,^53–55^ supports initiating EN within 24–48 hours of PICU admission, provided there are no contraindications such as bowel obstruction, severe shock, or high risk of aspiration. Perceived barriers to early EN include noninvasive ventilation, mechanical ventilation, large measured gastric residual volumes, vasoactive therapies, and extracorporeal life support. Delays in EN initiation have been linked to increased complications, including gut mucosal atrophy, bacterial translocation, and prolonged dependence on parenteral nutrition. Although existing studies are limited by residual confounding and underpowered RCTs, the overall trend and biological plausibility favor early EN. Current SCCM/American Society for Parental and Enteral Nutrition and European Society of Pediatric and Neonatal Intensive Care (ESPNIC) guidelines endorse early EN as the standard of care,^56,57^ underscoring that while stronger trials are needed, the best available evidence supports this recommendation.

##### Clinical Implications

PICU teams should assess enteral feeding readiness daily and implement standardized feeding protocols to facilitate initiation and advancement of early EN. Factors such as vasopressor use, mechanical ventilation, and gastrointestinal function should be considered, but enteral feeding should not be unnecessarily withheld in hemodynamically stable patients with signs of adequate perfusion. Strategies such as trophic feeding, postpyloric feeding, optimal bowel regimens to promote stooling, and the use of prokinetic agents may enhance successful initiation and advancement of EN.

## DISCUSSION

Prior recommendations identifying low-value practices and promoting high-value, evidence-based care in the intensive care unit have advanced the conversation around value-based care.^6,58^ However, most recommendations have been derived from adult critical care, where patient populations, physiology, and outcomes differ substantially; in pediatrics, recommendations must account for developmental heterogeneity, fewer baseline comorbidities, and unique risks such as long-term neurodevelopmental outcomes. Our study extended this work by using a pediatric-specific Delphi process to identify 5 key priority areas tailored to the PICU population, as outlined in Table 2.

**Table 2.** Summary of High-value Pediatric Critical Care Recommendations

Recommendation	Clinical Implication	Evidence Summary	Demonstrated or Potential Impact
Prioritize early mobilization therapy in the PICU	Developmentally appropriate mobility incorporated into daily care	Systematic reviews and multicenter studies show safety and functional outcome improvements	↓ Ventilation duration ↓ Delirium ↑ Functional recovery
Avoid reflexive cultures for isolated fever	Use diagnostic stewardship protocols to guide culture ordering	Diagnostic algorithms and multicenter quality improvement studies show reduced cultures and antibiotics without increased mortality	↓ Central line–associated blood stream infections ↓ Antibiotic use ↓ False positives
Perform daily extubation readiness assessments (ERTs)	Standardize ERT bundle and integrate spontaneous breathing trials into workflow	Randomized control trials and quality improvement initiatives show decreased IMV duration and extubation failure	↓ IMV duration ↓ Extubation failure ↑ Protocol adherence
Limit transfusions in stable patients with hemoglobin >7 g/dL	Apply restrictive transfusion strategies based on clinical stability	Large randomized control trials confirm safety and efficacy of 7 g/dL threshold in nonbleeding children	↓Transfusion exposure ↓ Costs ↓ Complications
Initiate EN within 48 h when feasible	Assess daily readiness and use standardized EN protocols	Cohort studies, randomized control trials, and meta-analyses support early EN benefits	↓ Infection ↓ Length of stay ↑ Gastrointestinal integrity ↑ Nutritional adequacy

There are limitations to our approach that should be explored. First, expert opinions in pediatric critical care medicine were used in select instances to overcome lower quality, nonrandomized clinical trial evidence. Second, the initial screening was based on input from selected members of the pediatric critical care medicine community with expertise or self-identified interests in safety and quality improvement, which may not accurately reflect the opinions of all actively practicing pediatric critical care clinicians. Third, selecting only 5 of the possible 30 topics may have overlooked important priorities that demonstrate strength in promoting higher value, evidence-based care.

Importantly, we believe this work can be a first step toward consistently incorporating evidence-based, high-value care into PICU clinical processes. Further, using improvement science and quality improvement methods can help measure compliance with implemented care practices, thereby providing more comprehensive evidence of the value and efficacy of consistently using expected care practices.

Identifying lower value practices is another key step in this journey toward value-based healthcare. However, adoption of evidence-based practices in medicine has been fraught with delays and failures, with the average time to adoption of new practices often exceeding 10 years from the initial evidence.^59–61^ Institutional barriers and legacy practices often support ingrained clinical practices.^62^ In contrast, regular reviews of evidence and the corresponding adoption of up-to-date practice can be difficult to institute and sustain over time due to change fatigue, skepticism, and a failure to build a compelling case for change. Although we acknowledge the relative paucity of pediatric-specific implementation science research to guide the adoption of these recommendations, we believe there is growing momentum suggesting that the collaborative blending of quality and implementation sciences can overcome these challenges.^7,63^

Furthermore, the unique characteristics of the pediatric critical care patient population pose barriers to adoption. With heterogeneity in the patient population ranging from newborns to adults, implementing a universal one-size-fits-all approach becomes challenging. Our recommendations target standardized practices that often require not only system-wide integration but also multidisciplinary interprofessional collaboration and comprehensive education. In a team-based environment, the PICU requires multidisciplinary collaboration across care teams, including critical care physicians, nurses, respiratory therapists, and other specialty and subspecialty providers involved in care delivery. Integration into existing practices, such as legacy documentation, nursing workflows, and quality-tracking processes, can, for instance, pose additional barriers to adoption and implementation.

Expanding on this work, we note that important gaps remain to be addressed. As these recommendations are implemented, more robust research and evidence generation can help improve our understanding of how to avoid lower value care and promote evidence-based care to enhance patient outcomes across diverse populations. Research to explore strategies to improve goal-concordant care can identify and address implementation disparities. Through quality improvement methods and implementation science, PICUs should use methodologies such as Plan-Do-Study-Act cycles to iteratively refine interventions and develop clinical decision support tools that reinforce evidence-based practices in real time.^61,64,65^ Finally, adopting standardized practices to avoid lower value care and promote evidence-based care must be carried out in both existing pediatric critical care training programs and ongoing medical education. For example, using advancements in simulation-based training and/or artificial intelligence–driven machine learning can help clinicians adopt and integrate this into practice.

## CONCLUSIONS

Delivering high-value, evidence-based care in pediatric critical care is essential to improving patient outcomes while minimizing unnecessary interventions and resource overuse.^61^This review highlighted 5 key recommendations to optimize pediatric critical care, including limiting unnecessary imaging and laboratory testing, implementing early mobilization and EN, prioritizing tracheal extubation readiness assessments, and reducing inappropriate transfusions. These strategies are supported by strong clinical evidence demonstrating improved patient safety, reduced complications, and enhanced care delivery efficiency. By embracing these high-value care principles, pediatric intensivists can ensure that critically ill children receive the safest, most effective, and resource-conscious care possible. The widespread adoption of these recommendations has the potential to improve patient experiences, optimize healthcare use, and advance the standard of pediatric critical care. It is time to fully adopt these practices.

## Supplementary Material


