# Supplementary material for: Evidence-based Evaluation to Promote High-value Care in Pediatric Critical Care: It Is Time to Fully Adopt
Source: Pediatr Qual Saf. 2025 Dec 23;10(6):e863. doi: 10.1097/pq9.0000000000000863 (PMC13169143; doi:10.1097/pq9.0000000000000863)
Supplement: Supplementary file 1 [file pqs-10-e863-s001.pdf]

Supplemental Table: Original Thirty Recommendations Considered

Topic	Practices to Review
Diagnostics	Routine Culture Practices<br>EKG for QTC Monitoring<br>Daily CXR for Intubated Patients<br>Routine Electrolyte Testing and Monitoring<br>Routine or Scheduled Urinalysis Testing<br>Routine Stool Guaiac Use<br>Inflammatory Marker Use of Antibiotic Duration<br>Telemetry Use
Therapeutics	Blood Transfusions<br>Respiratory Clearance Treatments<br>Avoidance of Prolonged NPO<br>Avoidance of Delirium Medications<br>Narrow Antibiotics Promptly<br>Routine Hypotonic Fluid Use<br>Routine GI Prophylaxis<br>Continuous Sedation and Neuromuscular Blockade<br>Steroid Use in Shock<br>Routine DVT Prophylaxis<br>Empiric Aspiration Pneumonia Coverage<br>Volume Overload Assessment<br>Plasma Exchange Indications
Care Processes	Discussion on Goals of Care<br>Interpreter Services<br>Progressive Mobility<br>Protocolized Approach to Sepsis<br>Multidisciplinary Rounds and Checklists<br>Isolation Precautions<br>Extubation Readiness testing<br>Daily Medication List Review<br>Delirium Screening
